# Strategies for Using Polydopamine to Induce Biomineralization of Hydroxyapatite on Implant Materials for Bone Tissue Engineering

**DOI:** 10.3390/ijms21186544

**Published:** 2020-09-07

**Authors:** Neha Kaushik, Linh Nhat Nguyen, June Hyun Kim, Eun Ha Choi, Nagendra Kumar Kaushik

**Affiliations:** 1 Department of Biotechnology, University of Suwon, Hwaseong 18323, Korea; neha.bioplasma@suwon.ac.kr (N.K.); jk8199@suwon.ac.kr (J.H.K.); 2 Plasma Bioscience Research Center/Applied Plasma Medicine Center, Department of Electrical and Biological Physics, Kwangwoon University, Seoul 01897, Korea; nhatlinhusth@gmail.com; 3 Laboratory of Plasma Technology, Institute of Materials Science, Vietnam Academy of Science and Technology, 18 Hoang Quoc Viet, Cau Giay, Hanoi 100000, Vietnam

**Keywords:** polydopamine, hydroxyapatite, bone tissue generation, improved biomineralization, bio-implant materials

## Abstract

In the field of tissue engineering, there are several issues to consider when designing biomaterials for implants, including cellular interaction, good biocompatibility, and biochemical activity. Biomimetic mineralization has gained considerable attention as an emerging approach for the synthesis of biocompatible materials with complex shapes, categorized organization, controlled shape, and size in aqueous environments. Understanding biomineralization strategies could enhance opportunities for novel biomimetic mineralization approaches. In this regard, mussel-inspired biomaterials have recently attracted many researchers due to appealing features, such as strong adhesive properties on moist surfaces, improved cell adhesion, and immobilization of bioactive molecules via catechol chemistry. This molecular designed approach has been a key point in combining new functionalities into accessible biomaterials for biomedical applications. Polydopamine (PDA) has emerged as a promising material for biomaterial functionalization, considering its simple molecular structure, independence of target materials, cell interactions for adhesion, and robust reactivity for resulting functionalization. In this review, we highlight the strategies for using PDA to induce the biomineralization of hydroxyapatite (HA) on the surface of various implant materials with good mechanical strength and corrosion resistance. We also discuss the interactions between the PDA-HA coating, and several cell types that are intricate in many biomedical applications, involving bone defect repair, bone regeneration, cell attachment, and antibacterial activity.

## 1. Introduction

**Figure 1:**
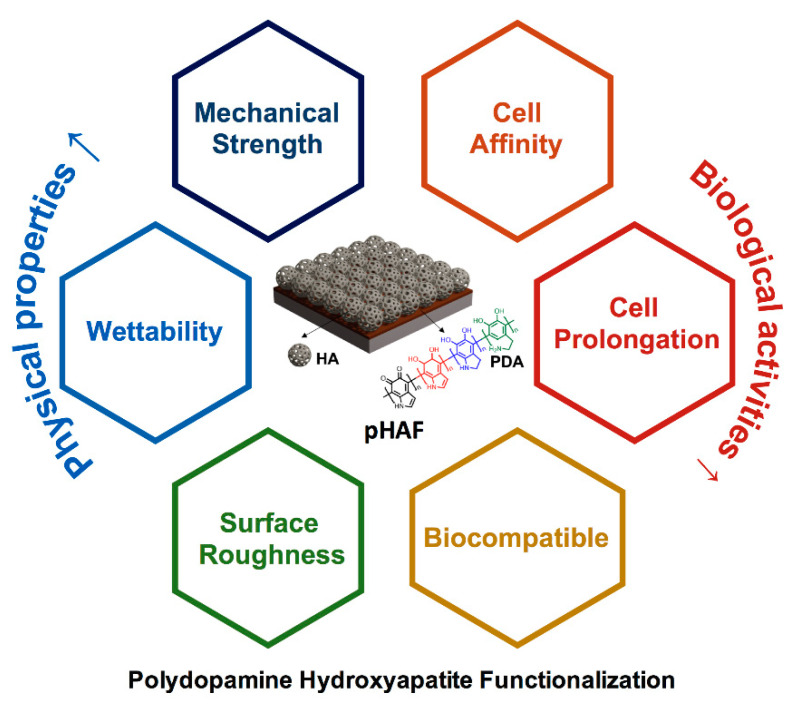
Polydopamine Hydroxyapatite Functionalization (pHAF) approach to improving the physical properties and biological properties for bone tissue engineering materials.

In the field of bone tissue engineering, designing the appropriate material for implantation is a vital aspect, which requires a comprehensive understanding of material composition, physical properties, structures to mimic the properties of the natural human bone. Also, other important factors, such as biocompatibility, cellular affinity, and growth of new bone tissue on the applied materials, should be thoroughly considered. One of the main components of the natural bone is hydroxyapatite (HA), a calcium phosphate crystal derivative that is hierarchically arranged between the collagen fibrils. During the last few decades, scientists have great attention to the utilization of HA for bone tissue engineering research, as well as practical applications [1]. Based on the polydopamine (PDA) coating methods, Ryu et al. developed an unprecedented method to mineralize HA on various types of substrates [2]. The polydopamine-hydroxyapatite functionalization (pHAF) method is a rapid and easy approach for functionalizing less-bioactive materials with HA, thus significantly improving their properties for the practical application in bone tissue engineering. This strategy can serve as a modifier on the various bone substrates to expand their interfacial properties that could provide substantial possibilities for tissue constructs. Similar to traditional approaches with regard to surface modification or biomolecule immobilization, the PDA-assisted surface functionalization demonstrates exceptional features, comprising mechanical strength, stable performance, and flexible applications. Though the detailed mechanism behind the formation of the PDA coating layer on the material substitute is still controversial, the PDA-HA assisted surface modification is expanding its applications in osteogeneration. Over the last 10 years, this unique method has gained much attention from researchers, with many publications on a wide range of implant materials, including metals, ceramics, polymers, to improve both their physical properties and biological activities (Figure 1). Herein, this review exclusively concentrates on the current advances of the pHAF method for bone tissue engineering with concerns of bone defect repair and antibacterial resistance.

## 2. Polydopamine Hydroxyapatite Functionalization

### 2.1. Polydopamine 

**Figure 2:**
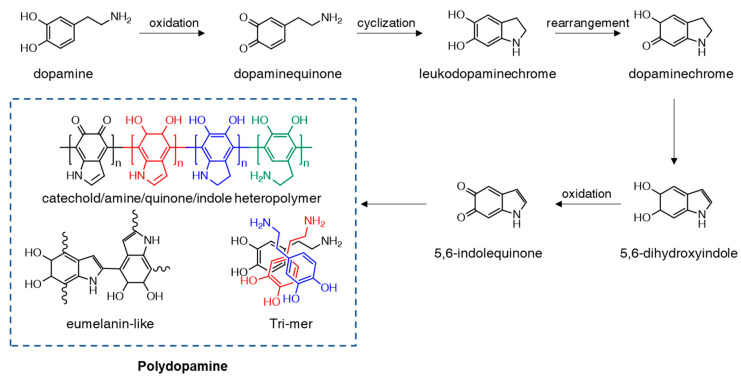
Schematic illustration of the polymerization of dopamine into polydopamine under the alkaline condition.

This observing natural phenomena has always been a principal foundation for innovative discoveries and inventions throughout the history of human science. The term “biomimetics” refers to the imitation of natural structures, designs, and mechanisms to benefit humanity. The marine blue mussel Mytilus edulis is a notable case study of biomimetics due to its robust adhesive properties for attaching to virtually all surface types. The byssus of the mussel is comprised of adhesive proteins Mefp3 and Mefp5. These two compounds contain an abundant amount of 3,4-dihydroxy-L-phenylalanine (DOPA), lysine, and histidine residues, resulting in high byssus adhesiveness. Inspired by this important blue mussel feature, the synthetic polymer polydopamine (PDA) was first introduced by Lee et al. in 2007 and has become one of the most prominent biomimicry materials [3]. PDA thin film was directly coated onto a substrate material through a self-polymerization process of dopamine (DA) moiety in water, with a pH >7.5. The PDA formation mechanism consists of several complex steps that remain a subject of debate (Figure 2) [4,5]. In a general chemical preparation with an alkaline solution, the DA molecule can be oxidized to dopaminequione, which then undergoes intramolecular cyclization into leucodopaminechrome. Subsequently, leucodopaminechrome is easily oxidized and rearranged to form 5,6-dihydroxyindole and 5,6-indolequinone. All the catechol, quinone, and indole products formed from the abovementioned reactions are considered the main building blocks of the PDA structure. Besides the commonly used chemical synthesis, other studies have presented alternative approaches for PDA coating via different methods such as enzymatic-catalyzed, electrochemical deposition, UV irradiation, and plasma-assisted deposition [4,6]. Noteworthy, besides PDA’s adhesion properties, it is also a biocompatible material with high-cell affinity that is used in biomedical applications. The catechol and amino groups on the PDA structure have strong interaction with the phosphates and choline groups of the cell membranes [7,8,9]. Based on these advantages, PDA-functionalized materials have proven to be efficient in cancer therapy and other intercellular applications [10,11,12,13]. Besides, PDA’s antimicrobial, soft tissue engineering, and drug delivery capability have also been reported in detail [14,15,16].

### 2.2. Hydroxyapatite Biomaterials and Biomineralization

Bone and teeth are the hardest substances found in vertebrates. They consist of several biominerals, which are biogenic hierarchical structures with unique properties and morphologies. Biominerals exist in crystalline, paracrystalline, and amorphous phases; they contain several inorganic constituents, such as carbonates, phosphates, halides, sulfates, etc. Naturally, biominerals are formed through a process called biomineralization, which involves the combination of biomacromolecules such as proteins, carbohydrates, lipids, and amino acids inside the living organism. Biomaterials have been widely used in two main areas, so-called implantable prostheses and scaffold tissue engineering [17]. The purpose of synthetic implants is to replace or support lost and damaged tissues; as a substitute, implanted devices remain within the living system constantly or temporarily.

Hydroxyapatite (Ca_10_(PO_4_)_6_(OH)_2_ or HA) is a calcium-phosphate (CaP)-based material. HA is one of the most prominent biomaterials used for bone substitution due to the similar chemical constitution and crystallographic structure of bones and teeth. HA is chemically stable in physiological conditions and possesses adequate bioactivity and biocompatibility. Nevertheless, bulk HA demonstrates inferior mechanical properties compared to natural bones. The HA structure contains both micropores and macropores that could affect the tensile strength and fatigue resistance, thus affecting the direct application of HA as a bone implant. Therefore, the utilization of HA with additive materials has attracted great attention [18]. In this scheme, the coating of HA on a stronger material is a favorable approach for practical application. For instance, metal implants have superior mechanical strength, limited the growing and attachment of the osteoblast cell on their surface. A previous study of Godley et al. suggests that a bioactive bone-like apatite coating layer on the surface of a biomaterial is essential for bone formation on its surface [19]. Thus, functionalization of a metal surface with HA not only improves the mechanical properties but also enhance bioactivities such as cell adhesion, proliferation, migration, and differentiation [20,21,22,23]. The focus on the utilization of HA, Kokubo et al. demonstrated an in-vivo method for biomineralization of HA on the surface of materials, using a simulated body fluid, which has the cations and anions content closely to human body fluid [24]. This approach not only can be used to modify the surface of implant materials but also is considered as a tool for estimating the bone-bonding properties of those materials. Nevertheless, the mineralization of HA in the simulated body fluid (SBF) strongly depends on the material’s surface properties, which can limit the universal use of this method.

### 2.3. Functionalization Process

**Figure 3:**
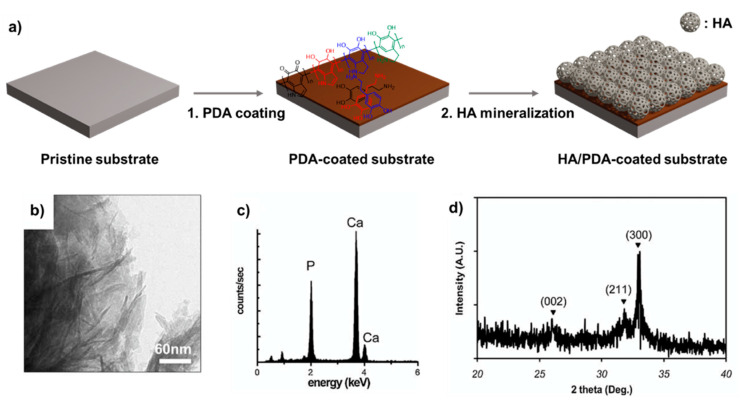
(**a**) Schematic illustration of polydopamine-assisted hydroxyapatite functionalization (pHAF) coating of the PDA layer by auto-oxidation of the DA on the substrate, followed by the mineralization of HA on PDA layer in SBF solution. Characterization of HA formed on the PDA-coated Titanium substrate: (**b**) TEM, (**c**) EDX, (**d**) XRD. Reprinted with permission from reference [2]. Copyright Wiley 2010.

Recently, a generic mechanism for biomimetic mineralization called pHAF was reported by Ryu et al. [2], utilizing PDA coating as a supporting layer for the growth of HA over a substrate material (Figure 3). As a multifunctional biocompatible material, PDA has recently gained considerable attention as a suitable solution to overcome the biomineralization of HA on the surface. Due to its potent bioadhesive capacity, PDA can improve the bioactivities and cellular behaviors of both implant and scaffold biomaterials. This approach involved 2 consecutive functionalization steps. First, a thin film of PDA was coated on the surface of the titanium substrate by submerging it in a DA solution in tris buffer pH 8.5, as previously described [3] Then, the coated material was transferred to a SBF [25,26]. The SBF was prepared with an ion concentration similar to human blood plasma, containing vital ions and anions such as Na^+^, K^+^, Ca^2+^, Mg^2+^, Cl^−^, HCO^3−^, SO_4_^2−^, HPO_4_^2−^ in tris buffer at pH 7.4. Then, the solution was incubated at 37 °C to promote the biomimetic mineralization of HA. The authors used PDA-coated titanium substrates to systematically study the reaction and HA formation mechanism. Briefly, under the SBF condition, CaP nucleation was occurred, forming the hemispherical CaP agglomerates on the PDA-coated Ti substrate during one day. After the second day, the agglomerates covered most of the substrate surface. The functionalization process was carried out for two weeks. After the functionalization, CaP crystal minerals were generated and fully covered the surface area of the PDA coated material. Noteworthy, the PDA coating layer not only played an anchoring role in binding Ca2+ cations onto the substrate but also cultivated the nucleation of HA biominerals. The morphological change suggests that the formation of CaP biominerals onto the PDA-coated surface is based on the layer-by-layer growing model due to the strong affinity of CaP with the PDA moieties. Several physical characterizations, such as Transmission electron microscopy (TEM), X-ray diffraction (XRD), energy-dispersive X-ray spectroscopy (EDX) were carried out, showing the CaP biomineral was HA biomineral (Figure 3). Interestingly, the pHAF approach can be applied to a variety of materials, including metals, ceramics, semiconductors, and polymers. The HA-PDA composites showed strong adhesion stability under ultrasonication and peeling tests. The cellular response and toxicity test of the HA-PDA layer to preosteoblasts indicated that it was non-toxic to cells. Other reports emphasized that the pHAF coating can modify a non-bioactive surface into a bioactive one [27]. Also, the combination of PDA and HA demonstrates great potential in biomedical applications [28,29].

Interestingly, the formation of PDA and HA can also be achieved by employing an electrochemical approach [30,31]. For instance, Xie et al. presented a layer by layer assembly method using pulse electrochemical deposition (PED) to fabricate PDA-HA multilayer nanofilm on the surface of the Ti foil. [31] In the conventional pHAF mentioned above, the PDA and HA were fabricated individually. In PED, the formation of PDA and HA can occur alternatively in a single chemical bath by alternating the applied voltage. Both in vitro and in vivo studies showed that PDA-HA has high osteoconductivity and biocompatibility when applied. Not limited to the PDA thin-film structure, PDA nanospheres can play the role of template materials for mineralizing HA. The PDA nanospheres were synthesized via DA oxidation in a water-alcohol mixed solvent. Similar to thin-film PDA, the HA biomineral was easily generated on PDA nanosphere surfaces in SBF solution [32]. In another study by the same group, the biomimetic formation of HA on PDA spheres was accelerated using microwave radiation. The final product also showed good biocompatibility for improving cell attachment on HA-coated constructs. These hybrid spheres demonstrated the potential for an organic-inorganic structure for tissue regeneration [33].

## 3. Molecular Interaction of PDA or HA with Biological Cells

**Figure 4:**
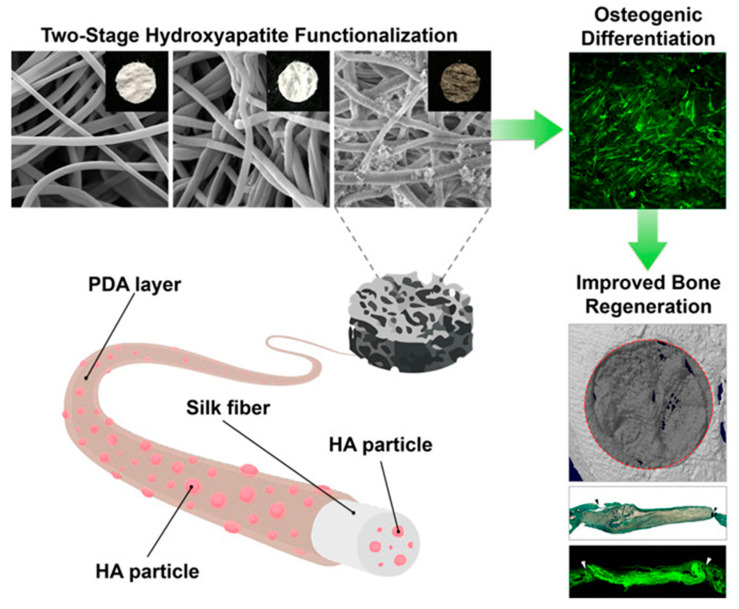
Schematic illustration and scanning microscopic images of the SF scaffold engineered with two-stage hydroxyapatite functionalization. Enhanced collagen deposition in critical-sized calvarial bone defects, eight weeks after human adipose-derived mesenchymal stem cell transplantation with two-stage hydroxyapatite-functionalized silk fibroin (SF) scaffolds and improved bone regeneration. Reprinted with permission from reference [43]. Copyright (2018) American Chemical Society.

The cellular response at the biomaterial–host tissue interface is predominantly critical in tissue regenerative medicine and bioengineering because it eventually dictates the outcome of implants in biological systems [34,35]. The biological interaction of apatite with cellular tissues is an essential characteristic of bone tissue regeneration. The conceptions in the mineralization and biological tissue interaction are varying due to the change in production skills, material nature, and size. Many researchers have been worked on a range of micro- and nano-surfaces with tunable properties to clarify the basic mechanisms intricate in biological cells–material substrate interactions and reveal the association between surface characteristics, including chemistry, stiffness, and cellular response (proliferation, differentiation, migration) [36,37]. As mentioned earlier, PDA is one of the most promising functional biomaterials developed in the last decade so far. It has been believed so far that the use of PDA is an easy and efficient method to regulate interfacial biological events in tissue engineering applications as a bioactive coating for direct signaling to cells [38,39]. It has been discovered that improving surface hydrophilicity could encourage cell spreading and attachment [40]. Surface roughness increased cell attachment owing to its increased protein absorption [41]. Moreover, an earlier study recommended that surface roughness at the micro- and sub-micro levels could facilitate osteoblast differentiation [42]. Interestingly, Ko et al. established a functional electrospun silk fibroin (SF) nanofibrous scaffold functionalized with two-stage HA particles, using PDA chemistry and fruitfully confirmed that HA over PDA coatings could promote osteogenic differentiation for enhanced bone formation in vivo and in vitro (Figure 4) [43]. To spread the amount of HA and help faster osseointegration, Xu et al. developed a HA coating using a biomimetic process assisted by PDA on the Hydroxyapatite/polyamide-66 (HA/P66) substrate. [44] Hence, the bioactive benefits of the surface roughness and HA coating could be the reasons for the HA-PDA-HA/P66 substrate supporting osteogenic differentiation of mouse mesenchymal stem cells, named as C3H10T1/2. This strategy successfully improves the capacity of biomaterials for better osteogenic induction as seen in vitro, as well as in vivo. This type of reconstruction of bone in vivo is closely correlated with the adhesion, proliferation, and differentiation of osteoblasts in vitro. The main reason that the HA coating promotes osteogenesis is possibly a combination of boosted hydrophilic improvement along with surface roughness and contact of more bioactive HA particles due to the presence of PDA. These landscapes may provide more osteoinductive and osteoconductive potentiating scaffolds for cellular protein binding, which promote osteoblast adhesion, proliferation, and differentiation. The strength of the interactions among PDA and templating molecules endure being examined in the future to be able to describe possible new templating vehicles. Notwithstanding such preceding fundamental studies, the accurate mechanisms that control how cells respond to PDA and HA or PDA-HA composite together still not fully elucidated so far and need to be investigated further. In over-all, the mechanism of action of a biomaterial is thought to be biocompatible, biotolerant, and bioresorbable. These modifications in understanding have followed, owing to the changes in the material properties and production methodology, and the deeper understanding of biomaterial interactions with the tissues.

## 4. Application of PDA-HA Functionalization for Implant Materials

### 4.1. Metals

#### 4.1.1. Titanium-Based Materials

Titanium (Ti)-based are the most commonly used metallic materials in biomedical applications due to their biocompatible and corrosion resistance properties, which are suitable for practical applications in bone tissue engineering [45]. An earlier report showed that the surface of Ti alloy could be improved by the pHAF method using PDA coating and the growth of HA in SBF. The PDA-coated Ti alloy showed better apatite formation ability than the pure Ti alloy, as demonstrated by HA formation followed with SBF immersion for 10 days, using scanning electron microscopy. It is worth to mention that the surface modification of the Ti alloy by coating with PDA did not alter the biological properties of the Ti alloy [46]. It has been proved that substrate-decorated PDA coatings could increase cell affinity and foster cell behavior as compared with bare substrates [47,48]. Hence, to enhance biocompatibility and bioactivity, several reports have focused on the growth and characteristics of PDA-assisted HA formation on titanium and its derivatives. In this regard, Zhe et al. invented an innovative idea to coat a porous anodized TiO_2_ layer using PDA to quickly immobilize HA on Ti-based substrates and measured its corrosive stability under normal saline conditions (0.9% NaCl), and reported the cell response of HA/PDA-coated anodized Ti surface (HAD-Ti) [49]. The HAD-Ti surface showed outstanding corrosion resistance (increased potentiodynamic polarization curves) and a strong affinity to water compared to the pristine surface. The in vitro test results demonstrated that incorporating HA on HDA-Ti promoted the adhesion of MC3T3-E1 cells, as visualized by higher expression of vinculin, an intracellular protein responsible for the focal adhesions to the actin cytoskeleton, in the cytoplasm of cells cultured on HDA-Ti surface and osteoblast precursor alkaline phosphatase (ALP) activity (~12%). To enhance the deposition rate of HA on PDA-coated Ti substrate, Wu et al. presented a new approach implementing casein phosphopeptide (CPP) [50]. A bilayer of PDA/CPP was prepared on Ti surface via a two-step immersion coating process. The outer layer of the CPP acted as a template to bind Ca^2+^ to promote nucleation and growth of HA in SBF. The PDA/CPP worked as the supporting template that enhanced binding between the HA and Ti substrate. Moreover, the HA coating formation time on this substrate through the biomimetic HA coating method was less than one day, which was lesser as compared with the conventional pHAF method, which requires 14 days. To make the Ti more favorable for HA production, a facile and effective surface modification method based on DA polymerization, was developed by Chien and Tsai, to immobilize peptides, HA, and BMP-2 on Ti. Here, they combined Arg-Gly-Asp (RGD)-conjugated polymers, HA nanoparticles, and BMP-2 and mixed with an alkaline DA solution followed by immobilization on the titanium surfaces. They found that after seven days of culture, the cell number on Ti-DA/RGD/HA was about 4.42 × 10^4^ cells/cm^2^, higher than the Ti-DA/HA (2.65 × 10^4^ cells/cm^2^) only. Of note, cell proliferation was not much affected by the incorporation of HA nanoparticles. It is worth to mention that the improvement of cell adhesion and proliferation was largely due to PDA deposition and RGD incorporation but not HA nanoparticles alone. Elevated expression levels of BSP (bone sialoprotein), RunX2 (Runt-related transcription factor 2), ALP, OPN (osteopontin), and OCN (osteocalcin) further suggests the improved osteogenic differentiation. This cell adhesive surface modification technique presents a great perspective for developing orthopedics and tooth implants, through improving cell adhesion, osteogenic differentiation, and mineralization [51]. Maintaining the equilibrium between bone development and bone resorption, as well as decreasing bacterial infections, are two key challenges when using Ti as the implant in orthopedic surgeries. To overcome these challenges, Shen at al. prepared a PDA-assisted HA and lactoferrin (LF) multilayer structure (PDA-HA-LF) on the Ti surface using a biomimetic approach and spin-assisted layer-by-layer assembly method. Excessive concentration of LF was toxic to osteoblasts but has noticeable effects on growth inhibition of osteoclasts and bacteria (S. aureus and E. coli). Interestingly, cytotoxicity of LF was improved through PDA-assisted HA deposition. The modified Ti implant could greatly improve the proliferation (~20%) and differentiation (increased ALP activity ~1.5 fold) of osteoblasts after four days of culture [52] These modified Ti implants have the potential to regulate the balance amid bone resorption and bone formation, with an evident antibacterial effect. These Ti implant surfaces modified with LF peptide showed antimicrobial activity by also preventing biofilm growth [53].

In another study, Ti surface was fabricated with nanotopography through alkali-heat (AH) treatment, then its surface was further modified by dipping substrates into the aqueous DA solution based on its self-polymerization [54]. HA was added to improve soft tissue attachment around the transcutaneous implant, and carboxymethyl chitosan (CMCS) was added to enhance its antimicrobial activity. The results indicated that the PDA-modified AH-Ti surface was a superior substrate for human gingival fibroblast (HGF) adhesion, spread, and proliferation. They showed that AH-Ti-PDA-HA and AH-Ti-PDA-CMCS were slightly (~5%) higher than AH-Ti-PDA at five days, indicating that the incorporation of HA and CMCS could further promote cell proliferation. Notably, AH-Ti-PDA-HA-CMCS had the maximum (~20%) increase in HGF cell proliferation with adhesion (~12%) properties and FN absorption (~15%). The addition of CMCS provided the surface with a more effective antibacterial effect. This suggested that combining both improved soft tissue integration and antibacterial properties could be achieved by using PDA-modified AH pre-treated Ti implants, which has huge potential in optimizing dental implant design.

**Figure 5:**
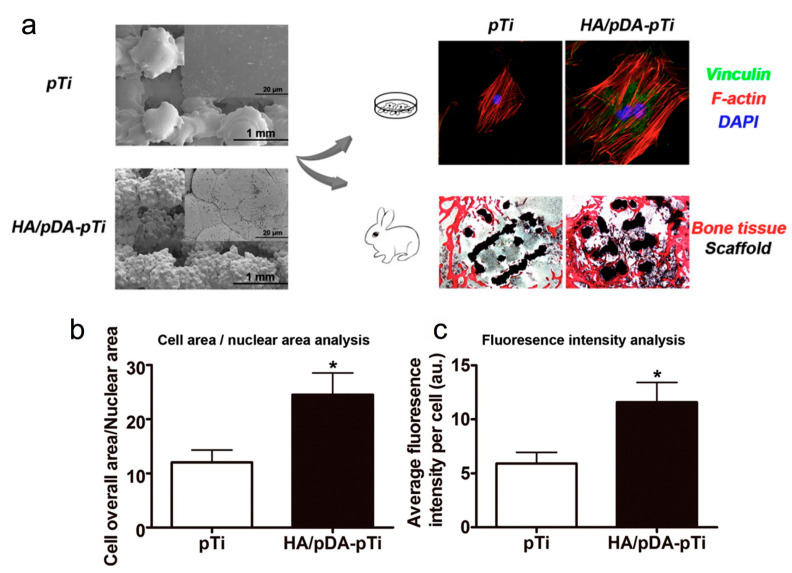
(**a**) Scanning electron microscopic (SEM) images of bare porous Ti6Al4V scaffolds (pTi) and polydopamine-assisted hydroxyapatite coating on titanium surfaces (HA/pDA-pTi). Fluorescent staining of MC3T3-E1 cells adhered to pTi and HA/pDA-pTi scaffolds, and (**b**) analysis of morphology of SEM images as shown in panel a and (**c**) fluorescence intensity of vinculin staining for cells on different scaffolds. Asterisks (*) indicate statistical significance compared to the pTi group, *p* < 0.05). Reprinted with permission from reference [55]. Copyright (2015) American Chemical Society.

Besides bulk Ti materials, porous Ti scaffolds are receiving much attention due to their unique advantages of high surface area and durable mechanical strength. Additionally, the porous structures could promote chemical diffusion, thus enabling the freshly formed bone to grow into the scaffolds. Since the amendment of the scaffold surfaces, predominantly inner surfaces, is important to improve the osteointegration of these scaffolds, Li et al. employed a biomimetic procedure to construct HA/PDA onto porous Ti6Al4V scaffolds (pTi) fabricated using electron beam melting method [55]. They stated that the attachment and proliferation of mouse preosteoblast cells (MC3T3-E1) were approximately 25% enhanced by the HA/PDA coating on the scaffold surface as compared to the non-modified surfaces. MC3T3-E1 grown on the HA/PDA-coated Ti6Al4V scaffolds showed upregulation of osteogenic genes, such as Runx2 (~1-fold), ALP (~1-fold), OCN (~1-fold), OPN (~1.5-fold), and collagen type-1 (~1.5-fold) compared with bare Ti6Al4V scaffolds after 14 days of culture. Moreover, in vitro studies revealed that HA/PDA coating on surfaces of porous Ti6Al4V scaffolds boosted osteointegration and endorsed bone regeneration in rabbit femoral condylar defects after implantation for 12 weeks by approximately one fold (Figure 5) [55].

#### 4.1.2. Magnesium-Based Materials

Besides titanium, researchers verified the use of magnesium as a conceivable biodegradable metal for forming orthopedic implants. However, the use of magnesium-based biomaterials remains the biggest challenge in orthopedics due to its high degradation rate in physiological environments. To overcome this challenge, by minimizing magnesium degradation and improving its biocompatibility as an implant, Feng et al. developed a method where micro-arc oxidation coating doped with HA particles (MAO-HA) was applied as the inner coating, and the PDA film was made by DA self-polymerization as the outer coating [56]. The key outcome was that the addition of PDA coating transformed the surface wettability from hydrophobic to hydrophilic. The water contact angle of MAO-HA coating is measured to be 100.5°, while that of PDA/MAO-HA coating is 80.1°, which indicates that the addition of PDA has shifted the surface wettability from hydrophobic to hydrophilic. In vitro experiments showed that the PDA/MAO-HA coatings revealed better corrosion resistance, with the corrosion current density declining from 2.09 × 10^−5^ A/cm^2^ to 1.46 × 10^−6^ A/cm^2^. The cell-surface interaction experiment showed that the PDA/MAO-HA coating exhibited good biocompatibility with increased cell proliferation (~1.2-fold at day 5), and cell adhesion properties as seen by scanning electron microscopy. This strategy endows magnesium alloys with corrosion resistance and biocompatibility, thus could be considered as a potential option for magnesium alloy surface modification. Another group also described an approach using AZ31 magnesium alloys where an HA coating was prepared by PDA-induced biomimetic mineralization in a CaP solution to improve the in vitro corrosion resistance. Interestingly, their methods suggest that similar HA coatings on biodegradable AZ31 magnesium alloys could be achieved with a biomimetic strategy using PDA. These PDA/HA-coated AZ31 magnesium alloys also decreased the corrosion rate (1.82 × 10^−6^ A/cm^2^). Cytotoxicity assessment and morphology analysis observations also demonstrated that the modified AZ31 is not cytotoxic for L929 fibroblast cells, and promoted cell growth. AZ31/PDA/HA exhibited the ~12% largest number of cell proliferation as compared to the AZ31 surface. This method offers the possibility of using biomimetic PDA to arrange HA coatings onto the various magnesium alloys for corrosion protection [57].

#### 4.1.3. Calcium-Based Materials

Calcium is the main constituent of natural bone and the most abundant metal in the human body. Thus, using calcium-based materials for implantation is a great approach. In bone regeneration, calcium phosphates (CaPs) are extensively used for the preparation of biomaterials for hard tissue replacement and repair due to their similarities with the inorganic phase of the mineralized tissues of vertebrates. Considering the exceptional adhesive property of PDA, Liu et al. added PDA to calcium phosphate cement (CPC) to increase its compressive strength [58]. Specifically, PDA accelerated the conversion of dicalcium phosphate dihydrate and α-tricalcium phosphate into HA in the early stages. PDA-CPC was then soaked in SBF, thus promoting the rapid mineralization of a nanoscale HA layer onto its surface. The HA layer eventually acquired a large surface area, which beneficially influenced cell proliferation and proteins. This investigated method postulates a possible route for surface modification of CaP-based biomaterials. 

Calcium silicates (CS) is a notable family of osteoinductive biominerals used in bone generation and endodontic procedures [59,60]. They possess a superior sealing capability, which is an essential factor for repairing defective sites. The bone-bonding bioactivity of CS-based cement is generally determined by the formation rate of HA and other apatite-like biominerals. In this regard, some studies implemented the pHAF to modify the surface of CS-based cement. Recently, Wu et al. demonstrated the deposition method of PDA on hydrated tricalcium silicate cement (TCS) substrate via facile dip-coating in an aqueous DA solution [61]. TCS is another potential material that resembles the physical properties of natural human bone. The use of hydrated TCS substrate provides a continuous release of Ca(OH)2, which makes a favorable environment for DA self-polymerization. The corelease of Ca(OH)2 from the TCS substrate accelerated the covalent polymerization of DA, leading to faster deposition of PDA coating on TCS. Additionally, they showed that amorphous calcium phosphate (ACP), a precursor phase of bone-like HA, could quickly precipitate on the PDA-coated TCS surface within 5 min in SBF. They further extended their study to generate ACP on the surface of PDA-modified TCS. To this end, an ultrafast method was used to generate ACP on the surface of PDA-modified TCS through the kinetic deposition process within 5 min in SBF. This process is based on the fact that bidentate hydrogen bonds and electrostatic interactions produce a strong attachment amid a hydrated TCS surface and PDA coating. The mechanical study suggests that ACP is not thermodynamically stable and can be further transformed into HA. Despite not showing bioactivity in the final structure, this work provides a close insight into the formation of bone-like apatite on TCS via PDA modification, for future study on the development of this material [62]. Since ACP can be formed after PDA coating in bone formation that is converted into HA at the time of osteogenesis, Lin et al. proposed combining a polydopamine-polyacrylamide (PDA-PAM) single-network hydrogel and a PDA-PAM/ACP hydrogel consisting of ACP by blocking the extreme oxidation of DA [63]. These hydrogels have a high cellular affinity which confers a robust healing property. It was determined that the PDA-PAM/ACP hydrogels stimulate bone formation, evidenced by the high expression of ALP (~2.5-fold), Runx2 (~1.2-fold), and OCN (~<1-fold) involved in the osteogenic activity at 14 days of culture. Remarkably, 8% PDA-PAM/ACP gel efficiently repairs skull defects in vivo. This attempt could be a favorable approach for repairing skull defects using 8% PDA-PAM/ACP hydrogels.

Another family of calcium-based biomaterials that has drawn attention from researchers is calcium carbonates (CaCO_3_). Kim et al. synthesized CaCO_3_ crystalline microspheres by incorporating DA that was easily transformed into carbonated HA crystals when incubated in SBF at human body temperature [64]. Since carbonated HA is highly bioresorbable, they immersed them in SBF to evaluate their in vitro bone bioactivity as the assessment of the material’s osteoinductivity is typically performed by measuring the rate of HA production. It provides a new perspective for future treatment applications of bone degenerative diseases and bone defects.

### 4.2. Polymers

#### 4.2.1. Synthetic Polymers

Synthetic polymers, including poly(l-lactic acid) (PLLA), poly(glycolic acid) (PGA), polycaprolactone (PCL), are commonly used as replacements for natural bone due to their versatile properties. They have good biocompatibility, as well as high stability within the human body environment. Also, due to flexible properties, polymeric materials could be fabricated into on-demand shapes for specific applications. However, there are inevitable disadvantages of polymeric materials such as bioinert surfaces and weak mechanical strength. To improve these above-mentioned issues, several studies have focused on the functionalization of polymers with other bioactive molecules, including PDA-based coating [4,65].

**Figure 6:**
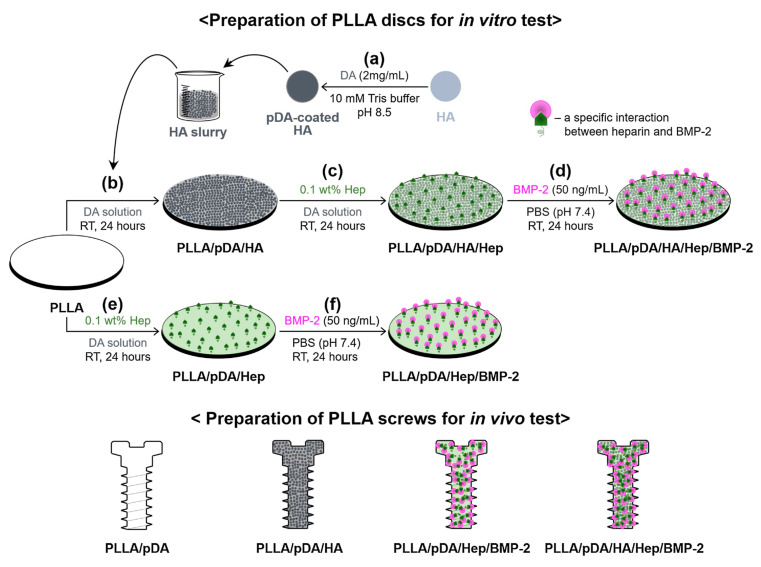
Schematic for the preparation PLLA/pDA/HA, PLLA/pDA/Hep/BMP-2, and PLLA/pDA/HA/Hep/BMP-2 disk and screw samples based on the pHAF method. Reprinted with permission from reference [66]. Copyright Elsevier 2018.

Among all, PLLA is the most used synthetic polymer for implantation due to high resistance to degradation and great tensile strength. However, this material possesses low wettability and cell adhesion, which are unfavorable for bone formation. To address these issues, Yun et al. performed the PDA-mediated HA, followed by functionalization heparin (Hep), and BMP-2 to improve the osseointegration of PLLA (Figure 6) [66]. The modified materials, so-called PLLA/PDA/HA/Hep/BMP-2, expressed elevated surface roughness (~48%) and wettability compared to pristine PLLA, thereby increasing the adhesion and cell proliferation (~1.2-fold at day 7) of the osteosarcoma (MG-63) cell line on the modified surface. Other vital parameters for bone generations, such as calcium deposition (~2.2-fold at day 21), OCN gene expression (~4-fold at day 21), and ALP activity (~0.5-fold at day 21) were also improved. An in vivo experiment performed on the rabbit tibia defect model was done to provide supporting evidence for the excellent osseointegration of PLLA/PDA/HA/Hep/BMP-2. Their findings suggest that the use of modified composite PLLA/pDA/HA/Hep/BMP-2 improved bone formation of the in vivo system at the interface. The bone volume (BV) and bone volume/tissue volume (BV/TV) ratio of PLLA/pDA/HA/Hep/BMP-2 was 3.18- and 3.21-fold greater than PLLA alone [66]. In addition to bulk PLLA, PLLA fibrous membranes were also subjected to pHAF to improve the osteoconductive activity. After the modification, the apatite structure was obtained on the surface of the electrospun PLLA fibrous membranes [67] Improved bioactivity for bone generation was also observed here. 

Copoly(phthalazinone biphenyl ether sulfone) (PPBES) is a polyarylether derivative that has gained much attention for implantation applications due to its properties, which are comparable to natural bone. Like most polymer-based implant materials, PPBES has a bioinert surface, which requires further functionalization for practical use. To address this issue, pHAF was conducted to form an apatite-coated layer on the PPBES substrate to enhance its cytocompatibility. Apatite coating on PPBES exhibited features of particles as similar to that produced from HA. The modified PPBES demonstrated low cytotoxicity (almost negligible) to NIH-3T3 cell lines, while cell adhesion was increased by 1.2-fold as detected by scanning electron microscopy. The results open a new approach for surface modification of poly(aryl ether) derivatives to extend their application in bone tissue engineering [68]. The pHAF was also applied in a PCL scaffold to obtain highly osteoinductive implantation composite (PCL-PDA-HA). Compared to PCL, the resulting scaffold demonstrated superior mechanical properties such as tensile strength and Young’s modulus. The 3D microstructure of the PCL-PDA-HA scaffold was suitable for the survival and differentiation of stem cells. The resulting scaffold was used together with the injection of substance P (SP) to increase bone tissue growth. Thus, this in situ bone regeneration strategy, which combines the recruitment of endogenous stem cells from the bone marrow to defective sites, and implantation of a highly biocompatible and osteoinductive cell-free scaffold system, has potential as an effective therapeutic in regenerative medicine. This work demonstrates that an effective strategy employs the mobilization of stem cells to the injury site, and is supported by a bioactive implant material to promote bone generation [69].

**Figure 7:**
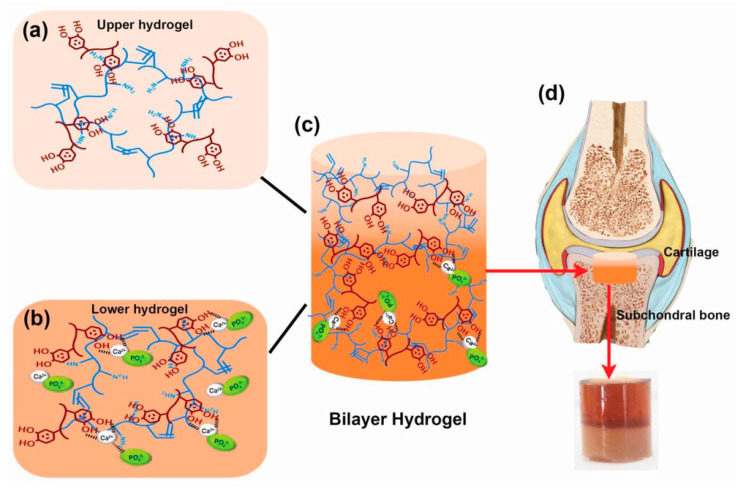
(**a**) The upper hydrogel layer for cartilage defect repair was prepared by mixing Gelatin methacryloyl (GelMA) with polydopamine (PDA) (**b**) The prepared lower layer hydrogel for subchondral bone repair was prepared by mixing Ca^2+^-GelMA with PO_4_^3−^-GelMA to generate hydroxyapatite (HA). (**c**) Combination of these two hydrogels to form the final structure of the hydrogel bilayer. (**d**) Illustration of the application of the hydrogel bilayer for the bone defect repair. Reprinted with permission from reference [71]. Copyright © 2019 WILEY-VCH.

Apart from designing the implant material for bone formation, the treatment of infected bone defects remains a tough clinical challenge. Therefore, the design of implants with osteogenesis and anti-bacterial activity is extremely important for bone healing with infection prevention. Consequently, Wei et al. fabricated bioresorbable porous-structured microspheres from an amphiphilic block copolymer consisting of PLLA and poly (ethyl glycol) blocks [70]. Followed by PDA surface coating, the microspheres were loaded with nanosilver via the reduction of silver nitrate and HA using the biomineralization process. Interestingly, these synthesized microspheres showed no negative effects on the differentiation of bone marrow mesenchymal stem cells regardless of strong antibacterial activity as seen in in-vitro studies. However, when bone defects (*ϕ* = 8 mm) were initiated using S. aureus bacteria, the dual-purpose microspheres proved an effective approach for killing bacteria in the rat cranium and at the same time stimulating new bone formation efficiently as observed by the higher expression of osteogenic genes OPN (~5%), OCN (~4%), and collagen type-1 (~8%) at day 14. These developed dual-purpose microspheres were biodegradable and potentially injectable, with both osteoconductive and antibacterial activities for treating infected bone defects. Since repairing cartilage defects is also another important aspect of bone tissue engineering, soft polymeric materials such as hydrogels are ideals candidates for this application. Incorporating hydrogels with biominerals can increase the generation of new bone cells in the defective sites. In this regard, Gan et al. functionalized a gelatin methacryloyl hydrogel (GelMA) by the pHAF method to form a bilayer structure [71]. In this method, the upper layer consisting GelMA-PDA hydrogel served as a cartilage repair layer, whereas the lower gelatin methacrylamide-polydopamine/calcium phosphate (GelMA-PDA/HA) hydrogel worked as a subchondral bone repair layer. To increase the efficiency of hydrogels towards tissue inducibility, transforming growth factor-beta 3 (TGF-β_3_) was immobilized in the upper layer to stimulate cartilage regeneration. Also, BMP-2 was immobilized in the lower layer to induce bone redevelopment (Figure 7) [71]. Besides improved mechanical strength, the swelling ratio (~70% reduction from GelMA hydrogels) and the stability of the GelMA-PDA/HA bilayer hydrogel were improved. This hydrogel enhanced the adhesion and differentiation of bone mesenchymal stem cells in vitro. In addition, the GelMA-PDA/HA was also tested in vivo for knee joint defect reparation in rabbits, showing higher subchondral bone formation compared to pristine GelIMA. They observed that the subchondral bone regeneration capacity of bilayer hydrogels was 20% higher compared to pure GelMA hydrogels, and the incorporation of growth factors in bilayer hydrogels further improved bone tissue regeneration by 20%.

#### 4.2.2. Natural Polymers

For the last several years, HA and collagen have been broadly used for coating metallic implants to facilitate osseointegration. Most of these methods for coating HA are not cost-effective due to high temperature and energy power requirements, while in the case of collagen coating, the available methods produce unstable coatings. With these obstacles in mind, Tapsir et al. used the PDA film as an intermediate layer to immobilize collagen type I and HA on a medical implant made of stainless steel [72]. These implants were further biomineralized with HA in SBF for an additional seven days. They concluded that immobilizing collagen produced better HA formation and wettability properties that could be favorable for the attachment and proliferation of osteoblast cells on surgical implants. Moreover, the crystallinity and wettability properties were improved with the longest immersion time.

In bone tissue engineering, artificial implant biomaterials with distinctive constituents could present good biocompatibility and antibacterial performance. Although HA has good biocompatibility and bone generation, it has little effect on microbial/bacterial growth when used clinically. Therefore, to ensure and improve antibacterial activity, a PDA-assisted layer-by-layer assembly process was developed with a hybrid coating composed of HA, Ag nanoparticles, and chitosan (CS) fabricated on a Ti substrate [73]. This method revealed that these hybrid coatings could provide Ti implants with suitable antibacterial activity against *Escherichia coli* (63%) and *Staphylococcus aureus* (51.8%), as well as osteogenic activity, confirmed by high ALP activities (~20%). Of note, they mentioned that HA showed a weak antibacterial response, whereas the Ag-doped hybrid coating improved its antimicrobial effect by damaging the structure of E. coli and S. aureus cell strains more effectively. However, for bone regeneration applications, Ag^+^ is toxic to bone cells; the addition of CS efficiently reduces the cytotoxicity of hybrid coatings, which allows for long-term osteogenesis with significant cell growth. As mentioned above, layered osteochondral composite scaffolds are considered as a promising strategy for the treatment of osteochondral defects. Nonetheless, the lack of osseous support and incorporation of the subchondral bone layer often leads to the failure of osteochondral repair. Consequently, it is important to investigate new subchondral bone constructs tailored to maintain bone integration and healing. To address these challenges, recently Zhang et al. [74] developed a new strategy based on the novel three-dimensional porous biomimetic construct (HA/PDA-OTMS) by PDA regulating HA microspheres produced in the honeycomb-like mollusk shell-derived organic template (OTMS). Due to excellent biocompatibility, this biomimetic OTMS allowed the smallest material to generate a stable geometric space, providing elevated HA loading capacity. This 3D-construct composite offered suitable 3D support for osteoblast cell attachment, proliferation, and differentiation. It also showed the elevated levels of osteogenesis-related genes such as ALP (~2-fold), OCN (~4.5-fold), collagen type-1 (~4-fold) after 30 days of culture. These studies provide a good aspect of subchondral bone. 

### 4.3. Carbon-Based Materials

In recent years, carbon-based materials such as carbon nanotubes (CNT) or graphene have also gained great attention as new alternatives for bone implantation due to their great mechanical properties, such as high strength and lightweight [75]. For instance, graphene and its derivative reduced graphene oxide (RGO) have gained considerable attention as an alternative for implantation due to their lightweight and strong mechanical strength. Assessment of osteogenic differentiation and an increase in mechanical strength were the major advantages of incorporating RGO in polymeric-based matrixes [76]. With these advanced properties, graphene could be used as a supporting material, which can provide the stiffness for HA minerals. Nevertheless, the fabrication of graphene-HA composite structures remains a challenge. To overcome this, Liu et al. implemented pHAF methods to effectively generate HA on a reduced RGO surface [77]. Impressively, the HA biomineral generated by this method had a similar chemical composition to biological apatite in the human body. The hybrid RGO-HA demonstrated great biocompatibility with L929 fibroblast cells. These cells showed an increase in cellular proliferation by 120% (RGO-PDA) and 116% (RGO/HA), suggesting that these types of materials have the least cytotoxicity. With the combination of unique RGO and PDA-HA properties, this hybrid material possesses great potential, not only for implantation but also for other biomedical purposes.

Another notable carbon-based material that can be modified by pHAF for bone generation is CNT. Like graphene, adding CNTs could improve the mechanical strength of HA biominerals. Numerous studies indicated that using CNT-HA scaffolds could improve the flexibility and the strength to maintain osteoblast cells [78,79]. Lee et al. coated the CNT surface with 5 nm of PDA, thus enhancing the dispersion of CNT in the aqueous medium [80]. Similar results were observed in a study by Yan et al., which showed that the CNT-PDA maintained high dispersity in water for a few weeks after preparation [81]. Due to the improved dispersion, HA was then easily mineralized onto the CNTs’ surface using the SBF solution. The CNT-PDA-HA hybrid demonstrated lower cytotoxicity (63%) compared with pristine CNT (73%) when tested with osteoblastic MC3T3-E1 cells [80,81].

## 5. Conclusion and Future Prospectives

The broad use of PDA coating provides a simple one-step protocol that could be used for deposition on any substrate materials, regardless of material chemistry and dimensionality. The molecular structure, stability, conformity, and mechanical strength of the PDA coating in physiological environments make it more relevant to biomimetic mineralization. Herein, we postulated that pHAF is a favorable methodology to produce HA coatings on supportive structures such as metals, ceramics, polymers, and carbon-based materials. In addition to enhancing the mechanical strength, these material coatings with bone-like mineral deposits also deliver osteoconductivity with good bioreactivity, and, once integrated with appropriate biomolecules, osteoinductivity. Moreover, it was underscored that immobilization of cellular growth factors within the mineral deposit coatings could provide prolonged delivery of these biomolecules, thus supporting bone tissue regeneration [74]. With developments in these areas, it is expected that the PDA coatings could be broadly applied to biomedical, agricultural, and other areas. These properties of the HA coating increase its potential in clinical applications and improve the life span of implants. Considering that HA is favorably biocompatible and identical to the natural bone structure, the mussel-inspired approach for bone mineralization is a promising means for future applications in bone defects. Based on these advantages, we propose that these PDA-HA-based materials are encouraging potential candidates as coating materials for injectable scaffold materials and implants in bone tissue regeneration.

## Abbreviations

PDA	Polydopamine
HA	Hydroxyapatite
pHAF	Polydopamine-hydroxyapatite functionalization
SBF	Simulated body fluid
